# Case Report: Multidisciplinary peripartum management of diffuse capillary-malformation-like lesions with cerebrovascular malformation in pregnancy

**DOI:** 10.3389/fmed.2026.1889533

**Published:** 2026-07-22

**Authors:** ZhiGao Pei, YanFang He, ShuBo Zhang

**Affiliations:** Department of Anesthesiology, Affiliated Hospital of North China University of Science and Technology, Tangshan, China

**Keywords:** capillary malformation, central nervous system vascular malformations, cesarean section, multidisciplinary management, pregnancy

## Abstract

Diffuse capillary-malformation-like cutaneous lesions are uncommon during pregnancy, and their clinical implications become more complex when they coexist with focal neurological symptoms or cerebrovascular malformation. This case report describes a 25-years-old primigravida admitted at 36 + 6 weeks of gestation with diffuse congenital capillary-malformation-like skin lesions, a documented history of cerebrovascular malformation diagnosed 10 years earlier, obesity, and hypothyroidism. Physical examination revealed extensive dark red to violaceous patches over the face, trunk, back, buttocks, and extremities. Brain imaging showed mild volume reduction and sulcal widening in the left temporoparietal-occipital region, a left frontal ischemic lesion, and non-visualization of most of the left posterior cerebral artery, whereas magnetic resonance venography revealed no obvious abnormality. After multidisciplinary assessment by obstetrics, anesthesiology, neurology, neurosurgery, and endocrinology, the patient underwent elective cesarean delivery at 37 + 0 weeks under an individualized staged anesthetic strategy. Maternal oxygenation and hemodynamics were maintained within an acceptable range, fetal exposure to general anesthetic agents before delivery was minimized, and no perioperative neurological event occurred. A healthy female neonate was delivered with Apgar scores of 10 at 1, 5, and 10 min. This case illustrates that diffuse capillary-malformation-like cutaneous lesions may serve as a clinical clue prompting broader vascular and neurological assessment when they coexist with neurological symptoms, documented cerebrovascular malformation, or abnormal neuroimaging. It also highlights the importance of descriptive terminology, individualized peripartum risk assessment, and multidisciplinary planning under diagnostic uncertainty.

## Introduction

Congenital vascular anomalies comprise a heterogeneous group of disorders, and precise terminology is important for diagnosis, risk stratification, therapeutic planning, and scientific communication. Brain arteriovenous malformations are abnormal direct communications between arteries and veins without an intervening capillary bed, and they may present with headache, seizures, focal neurological symptoms, or hemorrhagic stroke (1). Their pathogenesis may involve abnormal embryonic vascular development and inherited vascular-anomaly syndromes.

In the present case, the patient had previously been labeled as having “congenital telangiectasia” or “congenital capillary hyperplasia” at an outside hospital. These terms are not standard diagnostic entities in current international vascular-anomaly nomenclature. Given the congenital onset and the widespread dark red-to-violaceous cutaneous patches involving the face, trunk, and extremities, we used the descriptive term diffuse capillary-malformation-like cutaneous lesions rather than assigning a specific syndromic diagnosis in the absence of genetic testing and specialist vascular-anomaly evaluation.

Isolated cutaneous capillary malformations are usually low-flow lesions and do not necessarily imply systemic hypercoagulability or deep vascular involvement. However, when diffuse capillary-malformation-like lesions coexist with prior focal neurological symptoms, a documented history of cerebrovascular malformation, or abnormal neuroimaging, clinicians should consider a broader vascular-anomaly spectrum, including capillary malformation-arteriovenous malformation syndrome, hereditary hemorrhagic telangiectasia, Klippel-Trenaunay syndrome, diffuse capillary malformation with overgrowth, and mixed vascular malformations with venous involvement (2–5). This uncertainty increases the complexity of obstetric and perioperative decision-making.

We report a pregnant patient in whom diffuse capillary-malformation-like skin lesions coexisted with cerebrovascular malformation, abnormal brain imaging, obesity, and cesarean-related prothrombotic risk. The educational value of this case lies not in the skin phenotype alone, but in the diagnostic reasoning and multidisciplinary pathway used to recognize potential cerebrovascular and thrombotic risks during late pregnancy and delivery.

## Case description

A 25-years-old woman, gravida 1 para 0, was admitted on 4 May 2026 at 36 + 6 weeks of gestation for planned delivery because of diffuse congenital capillary-malformation-like lesions, a documented history of cerebrovascular malformation, obesity, and hypothyroidism. Her height was 168 cm, weight was 104 kg, and body mass index was approximately 36.8 kg/m^2^. She had no history of chronic hypertension, diabetes mellitus, epilepsy, intracranial hemorrhage, or previous surgery. She had been hospitalized at approximately 32 weeks of gestation for fetal lung maturation.

Hypothyroidism was managed after endocrinology consultation with levothyroxine 12.5 mcg/day, with repeat thyroid-function testing recommended after 1 month. Laboratory assessment on 2 April 2026 showed hemoglobin 122 g/L and fibrinogen 6.61 g/L, without obvious abnormalities in the remaining coagulation profile. Additional findings included aspartate aminotransferase 11 U/L, total cholesterol 7.37 mmol/L, triglycerides 2.88 mmol/L, low-density lipoprotein cholesterol 4.71 mmol/L, and uric acid 465 μmol/L. On 4 May 2026, hemoglobin was 118 g/L, fibrinogen was 6.75 g/L, and D-dimer was elevated according to the local laboratory report.

**FIGURE 1:**
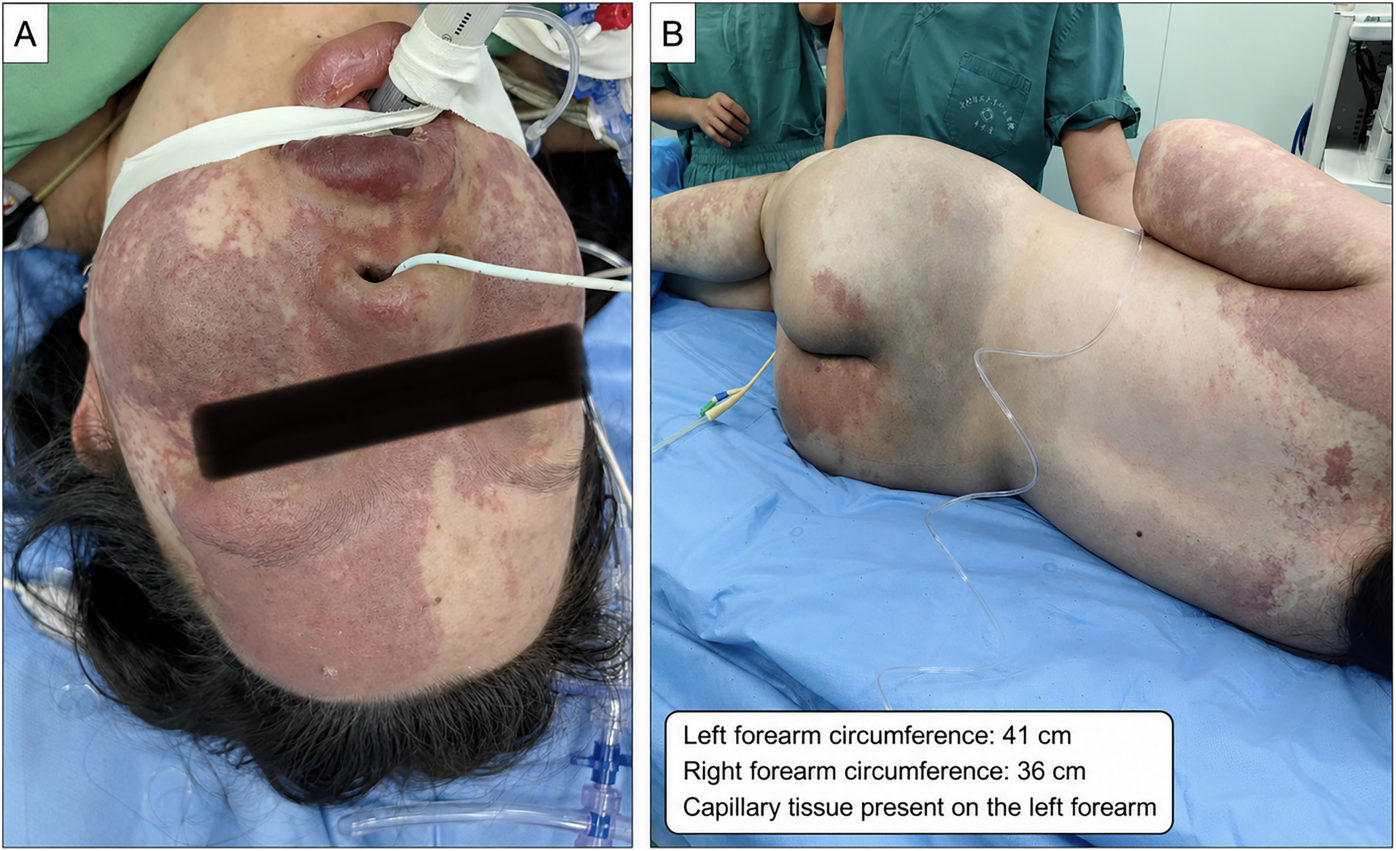
Clinical photographs showing diffuse capillary malformation-like lesions involving the face, trunk, and extremities. The left forearm was enlarged (circumference 41 cm vs. 36 cm on the right) with capillary tissue involvement. **A** represents the patient’s facial features, while **B** represents the patient’s body features.

Physical examination demonstrated extensive dark red to violaceous patches distributed over the face, trunk, back, buttocks, and extremities. Several lesions were confluent with irregular borders, but there was no ulceration, exudation, or active bleeding. In keeping with current vascular-anomaly terminology, these findings were described as diffuse congenital capillary-malformation-like cutaneous lesions. The clinical appearance of the cutaneous lesions is shown in Figure 1, and the timeline of key clinical events is summarized in Table 1. There was no known family history of vascular malformations, telangiectasias, cerebrovascular disease, recurrent unexplained bleeding, or hereditary vascular-anomaly syndromes. However, the absence of a known family history did not exclude a genetic or mosaic vascular-anomaly disorder because genetic testing was not performed.

**TABLE 1:** Timeline of key clinical events.

Time point	Clinical event and key findings
10 years before pregnancy	Right-sided limb numbness/weakness; diagnosis of cerebrovascular malformation at an outside hospital; original angiographic details unavailable.
Approximately 32 weeks	Hospitalization for fetal lung maturation.
2 Apr 2026	Endocrinology consultation; levothyroxine 12.5 mcg/day recommended. Hemoglobin 122 g/L; fibrinogen 6.61 g/L.
3 Apr 2026	Systemic screening: echocardiography showed left atrial enlargement; lower-limb venous ultrasound showed no thrombosis; abdominal ultrasound showed no definite visceral vascular malformation.
4 May 2026, 36 + 6 weeks	Admission for planned delivery with diffuse capillary-malformation-like lesions, documented cerebrovascular-malformation history, obesity, and hypothyroidism. Hemoglobin 118 g/L; fibrinogen 6.75 g/L.
Preoperative neuroimaging	Mild left temporoparietal-occipital volume reduction and sulcal widening, a left frontal ischemic lesion, and non-visualization of most of the left posterior cerebral artery; MRV showed no obvious abnormality.
Multidisciplinary assessment	Obstetrics, anesthesiology, neurology, neurosurgery, and endocrinology planned elective cesarean delivery with attention to blood-pressure control, airway risk, fetal exposure, and thrombosis surveillance.
5 May 2026, 37 + 0 weeks	Lower-segment cesarean delivery under staged individualized general anesthesia. Female neonate delivered 8 min after skin incision; Apgar scores 10/10/10; estimated blood loss 200 mL; no transfusion.
Postoperative day 1	Mother alert without neurological symptoms. Incisional pain score 0; ObsQoR-10 score 88/100. Neonate clinically stable.
Postoperative day 4	Mother and neonate discharged without clinically evident maternal neurological or thrombotic complications during hospitalization.
15 June 2026	Telephone follow-up approximately 6 weeks postpartum: mother reported no obvious neurological or thrombotic symptoms; infant was reported to be well.

MRV, magnetic resonance venography; ObsQoR-10, Obstetric Quality of Recovery-10.

Ten years earlier, the patient had presented to an outside hospital with right-sided limb numbness and weakness and had been diagnosed with cerebrovascular malformation. She had no confirmed history of intracranial hemorrhage, seizures, or impaired consciousness. During the current admission, she reported right upper-limb numbness with preserved active movement, but denied headache, dizziness, seizures, visual disturbance, dysarthria, limb weakness, or altered consciousness. Neurological examination revealed no definite focal deficit.

Brain magnetic resonance imaging at our hospital showed mild volume reduction and sulcal widening in the left temporoparietal-occipital region, a left frontal ischemic lesion, and non-visualization of most of the left posterior cerebral artery. Magnetic resonance venography showed no obvious abnormality. Taken together with the remote neurological symptoms, these findings were consistent with a congenital cerebrovascular developmental abnormality and/or previous ischemic change. Because historical angiographic data were incomplete, we did not attribute the prior neurological episode directly to thrombosis or to any specific vascular-anomaly syndrome.

**FIGURE 2:**
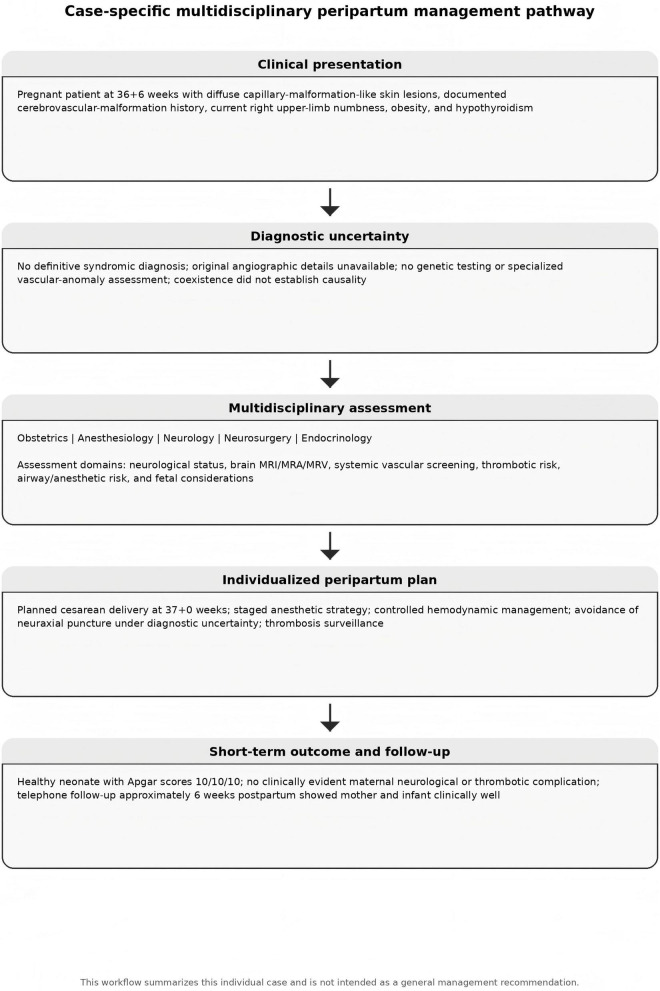
Case-specific multidisciplinary peripartum management pathway.

Given the diffuse cutaneous vascular phenotype and documented history of cerebrovascular malformation, systemic evaluation was undertaken before delivery. Echocardiography on 3 April 2026 showed left atrial enlargement with otherwise normal chamber dimensions. Venous ultrasonography of both lower limbs showed no evidence of thrombosis. Abdominal ultrasonography of the liver, gallbladder, pancreas, and spleen showed a moderately echogenic gallbladder-wall lesion, considered possibly a polyp, without definite evidence of visceral vascular malformation. These findings did not demonstrate deep venous thrombosis or abdominal solid-organ vascular malformation preoperatively; nevertheless, late pregnancy, obesity, and planned cesarean delivery justified careful venous thromboembolism surveillance. After discussion among obstetrics, anesthesiology, neurology, neurosurgery, and endocrinology, elective cesarean delivery at 37 weeks of gestation was planned. During multidisciplinary planning, there was no recent intracranial hemorrhage, no evidence of raised intracranial pressure, no progressive neurological deficit, and no acute neurosurgical indication. No absolute obstetric indication for cesarean delivery was identified. Vaginal delivery was considered; however, because of the documented history of cerebrovascular malformation, current right upper-limb numbness, abnormal neuroimaging findings, obesity, and incomplete characterization of the underlying cerebrovascular lesion, the team and patient favored planned cesarean delivery at 37 + 0 weeks. This approach was selected to allow controlled timing, close hemodynamic monitoring, coordinated anesthetic preparation, and immediate multidisciplinary availability. The case-specific decision-making process is summarized in Figure 2.

## Diagnostic assessment

The main diagnostic challenge was to determine whether the diffuse cutaneous vascular phenotype represented an isolated low-flow capillary malformation or a clue to a broader syndromic vascular anomaly with potential cerebrovascular involvement. The presence of widespread congenital lesions, a remote episode of focal neurological symptoms, a documented history of cerebrovascular malformation, and abnormal brain imaging argued against treating the skin findings as a purely cosmetic or isolated dermatological condition.

Differential diagnoses included capillary malformation-arteriovenous malformation syndrome, hereditary hemorrhagic telangiectasia, Klippel-Trenaunay syndrome, diffuse capillary malformation with overgrowth, and mixed vascular malformations involving venous components. Typical hereditary hemorrhagic telangiectasia was not supported by the absence of recurrent epistaxis, mucocutaneous pinpoint telangiectases on the lips, tongue, or fingertips, gastrointestinal bleeding, or a definite family history. Typical Klippel-Trenaunay syndrome was also not supported because there was no limb overgrowth, obvious varicosity, or documented extensive venous or lymphatic malformation. However, these conditions could not be fully excluded because genetic testing and specialized vascular-anomaly assessment were not performed.

The working diagnostic formulation was therefore: pregnancy with diffuse capillary-malformation-like cutaneous lesions, a documented history of cerebrovascular malformation, abnormal brain imaging suggestive of a cerebrovascular developmental abnormality and/or previous ischemic change, obesity, and hypothyroidism. This formulation was intentionally descriptive and risk-oriented, avoiding overdiagnosis while ensuring that cerebrovascular vulnerability and thrombotic risk were not overlooked. According to available outside-hospital information, spinal MRI performed before admission did not show obvious spinal vascular dilatation. Repeat spinal MRI was not performed during the current pregnancy, and the available evaluation was therefore insufficient to definitively characterize neuraxial vascular involvement. No spinal, epidural, or neuraxial vascular malformation was objectively documented during this admission, and coagulation testing did not identify a definite contraindication to neuraxial techniques.

## Therapeutic intervention

The patient underwent lower-segment cesarean delivery under general anesthesia on 5 May 2026 at 37 + 0 weeks of gestation. The perioperative plan focused on potential cerebrovascular events, hemodynamic instability, obesity-related airway risk, fetal exposure to anesthetic agents, and thrombotic risk. Electrocardiography, blood pressure, peripheral oxygen saturation, temperature, respiratory parameters, and anesthetic depth were continuously monitored. During the perioperative period, oxygen saturation was maintained at 98%–100%, temperature at approximately 36.4°C–36.9 °C, and heart rate at approximately 60–100 beats/min. Systolic blood pressure was approximately 180 mmHg on entry to the operating room, and subsequently decreased to a controlled range under anesthetic management. No persistent severe hypertension, hypotension, hypoxemia, or clinically significant arrhythmia was recorded.

The anesthesia and surgical teams adopted a staged strategy that had been planned preoperatively. After routine surgical disinfection and draping had been completed, the initial phase was conducted under intravenous analgesia-sedation with preserved spontaneous ventilation, rather than with the patient fully awake. Esketamine 20 mg (0.19 mg/kg) was first administered intravenously. After loss of the conjunctival reflex was observed, the obstetrician performed stepwise local infiltration of the abdominal wall using 40 mL of 1% lidocaine. A low-dose remifentanil infusion was administered for analgesia and sedation and was adjusted according to respiratory pattern, body movement, vocalization or moaning, and hemodynamic response. The clinical target of this phase was loss of the conjunctival reflex and absence of purposeful body movement, while maintaining spontaneous breathing and hemodynamic values within approximately 20% of baseline. Maternal discomfort was assessed clinically according to body movement, vocalization or moaning, respiratory pattern, and heart-rate or blood-pressure responses. Once adequate sedation and local anesthesia had been achieved, cesarean delivery was initiated. During skin incision and abdominal wall dissection, the patient maintained spontaneous ventilation without body movement, moaning, or other clinically evident signs of distress. After sufficient uterine exposure, anesthesia was deepened with propofol 150 mg (1.44 mg/kg), remifentanil 120 μg (1.15 μg/kg), and rocuronium 50 mg (0.48 mg/kg). A disposable laryngeal mask airway was then inserted by the anesthesiologist, while the obstetrician promptly opened the uterus, delivered the fetus, and clamped the umbilical cord. Anesthesia was subsequently maintained with propofol and remifentanil infusions titrated according to bispectral index monitoring. This staged approach allowed the initial incision and abdominal wall dissection to be performed under combined intravenous analgesia-sedation and local infiltration anesthesia, followed by rapid deepening of general anesthesia only after uterine exposure. The aim was to shorten the interval between deeper systemic anesthesia and delivery, reduce cumulative fetal exposure to anesthetic drugs before birth, and maintain controlled maternal hemodynamic conditions in the setting of documented cerebrovascular-malformation history and diagnostic uncertainty. This strategy was not intended as a generalizable anesthetic recommendation or as evidence of superiority over conventional general anesthesia; rather, it reflected individualized decision-making after multidisciplinary risk stratification.

Cesarean delivery proceeded uneventfully. A live female neonate weighing 2,870 g was delivered 8 min after skin incision, with Apgar scores of 10, 10, and 10 at 1, 5, and 10 min, respectively, and no neonatal intensive care was required. Mild transient uterine atony and minor incisional bleeding were successfully controlled with uterotonic therapy and topical hemostatic gauze. Estimated blood loss was approximately 200 mL, with no need for transfusion. After skin closure, liposomal bupivacaine 266 mg was infiltrated around the abdominal incision for postoperative analgesia. The case-specific multidisciplinary assessment, peripartum decision-making process, and short-term follow-up are summarized schematically in Figure 2.

## Follow-up and outcomes

After return to the ward, postoperative blood pressure was 129/68 mmHg. On postoperative day 1, the patient was alert and answered questions appropriately. She reported no headache, visual disturbance, limb weakness, altered consciousness, or new/worsening limb numbness. She was able to use the bathroom independently on the afternoon after surgery, although family assistance was recommended for safety. Resting and movement-related incisional pain scores were 0 on the Numerical Rating Scale. Movement-related visceral referred pain was 4, uterine contraction pain was 3, and sleep was not affected. The 24-h Obstetric Quality of Recovery-10 score was 88/100.

The neonate remained in the general ward with the mother. On postoperative day 1, neonatal respiration, responsiveness, and general condition were unremarkable. Formula feeding was provided temporarily because lactation had not yet started. The maternal postoperative course was uneventful, with no clinically evident neurological complication, thrombotic event, or wound complication during hospitalization. The patient was discharged on postoperative day 4. A telephone follow-up was performed approximately 6 weeks after delivery, on 15 June 2026. The mother reported no obvious neurological symptoms, including headache, seizures, altered consciousness, limb weakness, or new or worsening numbness. No clinically evident thrombotic symptoms, such as limb swelling, limb pain, dyspnea, or chest discomfort, were reported. The infant was also reported to be well.

## Discussion

This case report describes multidisciplinary peripartum management of a pregnant patient with diffuse capillary-malformation-like cutaneous lesions, a documented history of cerebrovascular malformation, abnormal brain imaging, obesity, and planned cesarean delivery. The favorable short-term maternal and neonatal outcome can be attributed to early recognition that the cutaneous phenotype might reflect a broader vascular-anomaly spectrum, systematic preoperative screening, individualized delivery planning, and coordinated perioperative monitoring. The case is noteworthy because it illustrates that diffuse vascular skin findings in pregnancy may serve as a clinical clue prompting broader vascular and neurological assessment in selected contexts, rather than being interpreted solely as isolated dermatological findings.

From a diagnostic perspective, avoiding non-standard terminology was important. The previous label of “congenital capillary hyperplasia” did not define the flow characteristics, depth, extent, or syndromic implications of the lesions. The descriptive term diffuse capillary-malformation-like cutaneous lesions preserved the visible phenotype while acknowledging diagnostic uncertainty. This approach is clinically useful because different vascular-anomaly syndromes have different implications for cerebral, pulmonary, hepatic, spinal, venous, and lymphatic involvement (2–5). In the present case, the absence of recurrent epistaxis, typical mucocutaneous telangiectasia, gastrointestinal bleeding, limb overgrowth, and obvious varicosity made classic hereditary hemorrhagic telangiectasia or Klippel-Trenaunay syndrome less likely, but did not eliminate the possibility of an atypical or incompletely characterized vascular-anomaly disorder.

The principal maternal concern was potential cerebrovascular vulnerability. Brain arteriovenous malformations may cause focal neurological deficits, seizures, or hemorrhagic stroke (1). Whether pregnancy and the puerperium substantially increase the risk of hemorrhage from brain arteriovenous malformations remains debated; however, recent studies suggest that the pregnancy-puerperium period may be associated with increased rupture risk and that management should be individualized (6–8). In this patient, the remote episode of right-sided symptoms, left frontal ischemic lesion, non-visualization of most of the left posterior cerebral artery, and incomplete historical angiographic data justified careful blood-pressure control, oxygenation, ventilation, and neurological surveillance.

Thrombotic risk required parallel consideration. Isolated capillary malformations do not usually cause localized intravascular coagulopathy or systemic hypercoagulability. Such phenomena are more typical of venous malformations or complex mixed vascular malformations. In this patient, no lower-limb thrombosis or abdominal visceral vascular malformation was identified preoperatively, and fibrinogen was not reduced. The elevated D-dimer and fibrinogen levels were interpreted cautiously in the context of late pregnancy, in which such changes are common and do not by themselves establish pathological thrombosis or localized intravascular coagulopathy. Therefore, thrombotic risk should not be attributed directly to the capillary-malformation-like lesions alone. Instead, risk assessment should integrate late pregnancy, obesity, cesarean delivery, perioperative immobility, and the possibility of an undetected complex vascular anomaly. Early mobilization, mechanical prophylaxis where appropriate, and vigilance for thrombotic symptoms were therefore rational components of care.

The cutaneous phenotype and the rationale for elective cesarean delivery should both be interpreted with caution. Diffuse capillary-malformation-like cutaneous lesions alone should not be considered an indication for cesarean delivery, nor do they by themselves contraindicate vaginal delivery or neuraxial anesthesia. Similarly, current evidence does not support routine cesarean delivery solely on the basis of a stable or unruptured cerebrovascular malformation, and mode of delivery should usually be determined by obstetric indications together with individualized neurological assessment. In our patient, there was no recent intracranial hemorrhage, no evidence of raised intracranial pressure, no progressive neurological deficit, no acute neurosurgical indication, and no absolute obstetric indication for cesarean delivery. Therefore, cesarean delivery should not be interpreted as mandatory for similar patients. In this case, the cutaneous findings served primarily as a visible clinical clue prompting broader assessment because they coexisted with a documented history of cerebrovascular malformation, current right upper-limb numbness, and abnormal neuroimaging findings. The management pathway should therefore be understood as a response to diagnostic uncertainty and combined patient-specific risk factors, rather than as a direct consequence of the cutaneous lesions themselves.

Vaginal delivery was considered during multidisciplinary discussion. However, because of the documented history of cerebrovascular malformation, current right upper-limb numbness, abnormal neuroimaging findings, obesity, and incomplete characterization of the underlying lesion, planned cesarean delivery was selected as an individualized approach to permit controlled timing, close hemodynamic monitoring, coordinated anesthetic preparation, and immediate multidisciplinary availability. This decision reflected patient-specific risk assessment under diagnostic uncertainty rather than a general recommendation. In selected patients with stable cerebrovascular malformations and favorable obstetric, neurological, and anesthetic assessments, vaginal delivery may remain appropriate.

The anesthetic strategy also requires cautious interpretation. Obstetric anesthesia literature emphasizes that neuraxial techniques are not universally contraindicated in parturients with intracranial pathology; the decision should instead depend on individualized assessment of intracranial pressure, mass effect, cerebrospinal-fluid dynamics, neurological status, coagulation status, airway risk, fetal considerations, and institutional expertise (9, 10). In this patient, staged general anesthesia was selected to avoid neuraxial puncture under diagnostic uncertainty and to facilitate controlled hemodynamic management, rather than as a response to a proven neuraxial contraindication. The absence of repeat pregnancy-period spinal imaging limits this reasoning, and the favorable Apgar scores and absence of clinically evident maternal neurological complications cannot establish superiority over neuraxial anesthesia, conventional general anesthesia, or other individualized approaches.

The strengths of this case include multidisciplinary evaluation, descriptive diagnostic framing, targeted systemic assessment, and short-term maternal and neonatal follow-up. The main limitations are the lack of original angiographic data from the neurological episode 10 years earlier, absence of genetic testing and specialized vascular-anomaly assessment, incomplete neuraxial imaging during pregnancy, and limited long-term maternal and neonatal follow-up. Therefore, the exact vascular-anomaly phenotype remains unresolved, causal links between the cutaneous and cerebrovascular findings cannot be proven, and favorable short-term outcomes should not be extrapolated to long-term neurological, thrombotic, vascular, or neonatal prognosis.

## Patient perspective

The patient expressed relief that both she and her baby recovered without major complications and appreciated the coordinated multidisciplinary care throughout delivery and early postpartum recovery.

## Conclusion

Diffuse capillary-malformation-like cutaneous lesions during pregnancy may warrant broader vascular and neurological assessment when they coexist with focal neurological symptoms, a documented history of cerebrovascular malformation, or abnormal cerebrovascular imaging. This case illustrates the value of descriptive diagnostic terminology, differential diagnostic consideration of syndromic vascular anomalies, targeted systemic assessment, peripartum thrombotic and hemorrhagic risk evaluation, and individualized multidisciplinary planning under diagnostic uncertainty. In the present patient, this strategy was followed by favorable short-term maternal and neonatal outcomes, although longer follow-up and genetic evaluation are needed to clarify the underlying vascular-anomaly phenotype and long-term prognosis. This case should not be interpreted as supporting routine cesarean delivery or routine avoidance of neuraxial anesthesia for all pregnant patients with diffuse cutaneous vascular anomalies or stable cerebrovascular malformations. In selected patients, vaginal delivery and neuraxial anesthesia may remain appropriate when obstetric, neurological, anesthetic, and imaging assessments are favorable.

## Data Availability

The original contributions presented in the study are included in the article. Further inquiries can be directed to the corresponding author.
